# A randomised evaluation of CollAborative care and active surveillance for Screen-Positive EldeRs with sub-threshold depression (CASPER): study protocol for a randomized controlled trial

**DOI:** 10.1186/1745-6215-12-225

**Published:** 2011-10-11

**Authors:** Natasha Mitchell, Catherine Hewitt, Joy Adamson, Steve Parrott, David Torgerson, David Ekers, John Holmes, Helen Lester, Dean McMillan, David Richards, Karen Spilsbury, Christine Godfrey, Simon Gilbody

**Affiliations:** 1 Department of Health Sciences, Seebohm Rowntree Building, University of York, Heslington, York, YO10 5DD, UK; 2 Centre for Mental Health Research, University of Durham, Durham, TS17 6BH, UK; 3 Leeds Institute of Health Sciences, Charles Thackrah Building, University of Leeds, 101 Clarendon Road, Leeds, LS2 9LJ, UK; 4 National Primary Care Research & Development Centre, Williamson Building, Oxford Road, University of Manchester, Manchester, M13 9PL, UK; 5 School of Psychology, Washington Singer Laboratories, University of Exeter, Perry Road, Exeter, EX4 4QG, UK

## Abstract

**Background:**

Depression accounts for the greatest burden of disease among all mental health problems, and is expected to become the second-highest amongst all general health problems by 2020. By the age of 75, 1 in 7 older people meet formal diagnostic criteria for depression. Efforts to ameliorate the burden of illness and personal suffering associated with depression in older people have focussed on those with more severe depressive syndromes. Less attention has been paid to those with mild disorders/sub-threshold depressive syndromes but these patients also suffer impairments in their quality of life and level of functioning.

**Methods/Design:**

The CASPER study has been designed to assemble an epidemiological cohort of people over 75 years of age (the CASPER cohort), from which we will identify those eligible to participate in a trial of collaborative care for sub-threshold depression (the CASPER trial).

We aim to undertake a pragmatic randomised controlled multi-centre trial evaluating the effectiveness and cost-effectiveness of collaborative care; a low intensity psychological intervention in addition to usual general practitioner care versus usual general practitioner care alone. General practitioners from practices based in the North of England will be asked to identify potentially eligible patients over the age of 75 years. Patients will be sent a letter inviting them to participate in the study.

We aim to recruit approximately 540 participants for the CASPER trial. A diagnostic interview will be carried out to ascertain trial eligibility with the major depressive episode module of the Mini International Neuropsychiatric Interview (M.I.N.I.), eligible participants randomised to either the intervention or usual care. The primary outcome will be measured with the Patient Health Questionnaire-9 (PHQ-9) and additional quality of life measures will be collected. Data will be collected at baseline, 4 and 12 months for both trial and cohort participants.

**Trial Registration:**

ISRCTN: ISRCTN02202951

## Background

### Problem to be addressed

Depression accounts for the greatest burden of disease amongst all mental health problems, and is expected to become the second-highest amongst all general health problems by 2020 [1]. By the age of 75, 1 in 7 older people meets formal diagnostic criteria for depression. Projected demographic changes mean that population strategies to tackle depression will increasingly have to address the specific needs of older people [2]. Amongst older people, depressive syndromes often affect people with chronic medical illnesses [3], cognitive impairment, social isolation or disability. Beyond personal suffering and family disruption, depression worsens the outcomes of many medical disorders and promotes disability [4]. Recently published National Institute for Health and Clinical Excellence (NICE) guidelines have acknowledged the symbiosis of physical health problems and depression [5,6]. The impairments in quality of life associated with depression are comparable to those of major physical illness [7].

Amongst older people, a clinical diagnosis of major depression is the strongest predictor for impaired quality of life (QoL) [7]. The focus has been on identifying and treating those with more severe depressive syndromes as set down in classificatory systems such as DSM IV [8] major depressive disorder or ICD 10 [9] moderate/severe depressive disorder [2]. UK policies under the Quality and Outcomes Framework (QOF) advocate screening for these threshold-level disorders amongst those with chronic physical health problems such as heart disease and diabetes [10]. Once detected, evidence-supported guidelines advocate the prescription of anti-depressant drugs and appropriate provision of psychological care [5,6,11].

Less attention has been paid to those with milder disorders/sub-threshold depressive syndromes or those who give positive responses to screening questions but do not have sufficient levels of depressive symptoms to meet diagnostic criteria [11]. A recent large cross-sectional study conducted in over 20 countries [7] showed that even relatively minor levels of depression are associated with a significant decrement in all QOL domains and with a pattern of negative attitudes toward ageing. Sub-threshold depression is also a clear risk factor for progression and the development of more severe depressive syndromes [12]. The focus of the current study will be in a population of screen-positive sub-threshold older adults.

### The need for a trial

Primary care services have increased their focus on screening for depression in older people. This screening programme has enabled primary care providers to identify and treat those with severe depressive syndromes. However, the screening programme also identifies those with sub-threshold depression. There is currently no clear evidence-based guidance regarding treatment for this patient group. The rationale for screening for depression in older people is clear, since a substantial portion of those with depression go unrecognised and untreated [11].

### Collaborative care for older adults

The vast majority of depression in older people is managed entirely in primary care, without recourse to specialist mental health services [2,11]. A range of individual treatments has been shown to be effective in the management of clinical depression in older people [11]. However, a repeated observation amongst those with depression has been the failure to integrate these effective elements of care into routine primary care services [13].

A new model of care has been introduced called Collaborative Care [14]. Collaborative care borrows much from chronic disease management and ensures the delivery of effective forms of treatment (such as pharmacotherapy and/or brief psychological therapy) and involves augmenting the role of non-medical specialists in primary care. The ubiquity of depression in primary care settings and the poor integration and co-ordination of care have led to the development and use of this model of care. Previous studies of collaborative care have found positive results [15-17].

In addition to the provision of collaborative care, low intensity psychological interventions, such as Behavioural Activation (BA), may benefit individuals experiencing depressive symptoms. BA focuses on the behavioural deficits common amongst those with depression and reintroduces positive reinforcement and reduces avoidance [18]. BA is about helping patients to '*act their way out*' of depression rather than wait until they are ready to '*think their way out*'. The effectiveness of this psychological approach is now well demonstrated [19]. BA can be readily delivered either over the phone by a trained case manager or face to face for those who experience difficulty using or accessing phone-based therapy [20]

### Limitations of previous trials

The major limitations of previous trials are two-fold. First, previous trials have generally included those with above threshold-level depression and have not looked exclusively at sub-threshold depression. Second, a key component of collaborative care is 'medication management' (encouragement of compliance and guideline-concordant prescription of anti-depressants) but anti-depressants are not indicated in those with screen-positive sub-threshold depression [5,6,11].

### Identifying depressive symptoms and validating measures of depression

**Figure 1:**
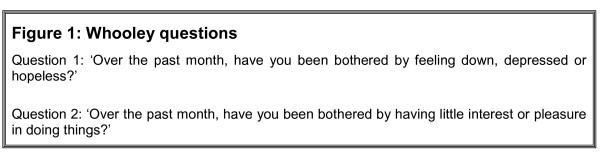
**The Whooley Questions**. The Whooley questions.

Two depression tools have been in regular use in primary care: the Whooley Questions [21], a brief 2 item depression questionnaire which has been used as a screening tool (the Whooley questions are detailed in Figure 1); and the Patient Health Questionnaire-9 (PHQ-9) [22] to measure depression severity once treatment is initiated. Both these tools have been adopted to fulfil QOF objectives in the UK (QOF DEP 1 and QOF DEP 2 respectively) [23].

A number of issues have been identified in relation to these screening tools. Firstly, neither tool has been validated in a UK elderly population. Where they have been validated, it has been against above-threshold depression and in non-elderly/non-UK primary care groups [24] or non-primary care populations [21]. Secondly, little is known about the ability of these instruments to identify less severe or sub-threshold levels of depression. Additionally, little is known about the significance of those who respond with positive answers to screening questions but do not have levels of depression that meet diagnostic criteria.

### Research objectives

1. To establish the clinical effectiveness of a low intensity collaborative care intervention for elderly people with screen-positive sub-threshold depression.

2. To examine the cost effectiveness of a low intensity collaborative care intervention for elderly people with screen-positive sub-threshold depression across a range of health and social care costs.

## Methods/Design

### The CASPER cohort

**Figure 2:**
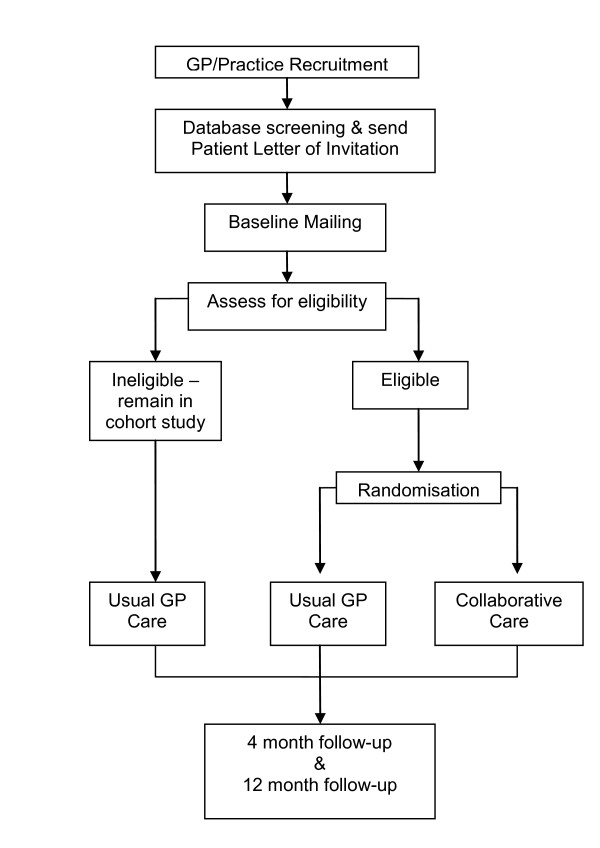
**Flowchart of the CASPER study**. Flowchart of the CASPER study.

The CASPER study has been designed to assemble an epidemiological cohort of people over 75 years of age (the CASPER cohort), from which we will identify those eligible to participate in a trial of collaborative care for sub-threshold depression (the CASPER trial; a flowchart of the CASPER study is detailed in Figure 2). It is anticipated that participants in the CASPER cohort will be given the opportunity to participate in other trials in the future, as part of a cohort multiple RCT (cmRCT) [25]. Participants for the CASPER study will be identified via GP practices only, no other facilities will be utilised to identify eligible patients. All patients who have been identified by the GP practice as eligible for an invitation mailing will be sent an invitation pack. Patients wishing to take part in the CASPER study will be asked to return completed consent and background information forms by post to the study centre. All consenting participants will then be asked to complete a baseline questionnaire. All participants who return valid baseline data will be included in the CASPER cohort. Inclusion in the CASPER trial is dependent on participants meeting the inclusion criteria and currently experiencing sub-threshold depression. This protocol describes the methods for identifying and recruiting all participants for both parts of the CASPER study (the epidemiological cohort study and the trial) and, specifically, the methods employed for the CASPER trial.

The trial has been designed as a multi-centre, unblinded, pragmatic randomised controlled trial lasting 45 months, comprising a 6 month 'pre-trial' period for refining the intervention, 12 month internal pilot trial period, 12 month definitive trial period, 12 month follow-up period and a final 3 month analysis period.

A qualitative evaluation will be carried out to examine the acceptability of collaborative care and BA for those over 75 and to ascertain the views of various stakeholders in order to assess the feasibility of delivering collaborative care and BA in the NHS. Additionally, an economic analysis will be carried out to assess cost-effectiveness.

### Identifying sub-threshold participants

**Figure 3:**
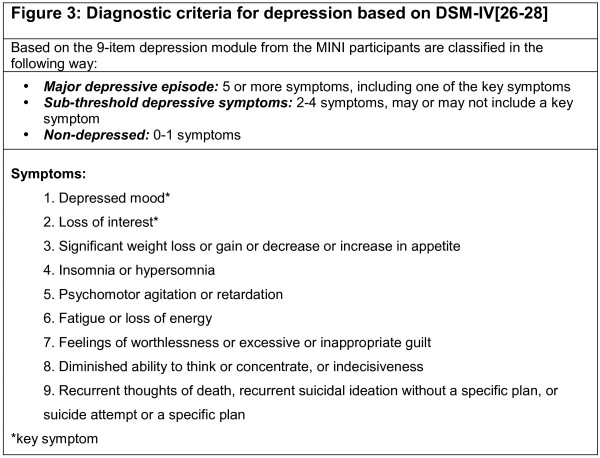
**Diagnostic criteria for depression based on DSM-IV**. Diagnostic criteria for depression based on DSM-IV.

Upon receipt of a valid baseline questionnaire all participants will be contacted by telephone to arrange a diagnostic interview (the diagnostic interview is carried out only once during the study); all diagnostic interviews will be carried out over the phone, by a trained researcher. The major depressive episode module of the Mini International Neuropsychiatric Interview (M.I.N.I.) will be used to ascertain the presence or absence of depressive symptoms and depressive disorders (sub-threshold depression and major depressive disorder) [26]. All participants diagnosed with sub-threshold depression will be randomised to either the intervention or control arm (see Figure 3[26-28] for the criteria). Participants diagnosed as either below- or above-threshold will not be randomised; participants will be advised of the outcome of their diagnostic interview and will be encouraged to remain in the study as part of the CASPER cohort and will be encouraged to return follow-up questionnaires. Participants diagnosed as experiencing a 'major depressive episode' on the M.I.N.I. will be referred to their general practitioner.

### The CASPER trial

Participants will be randomised into one of two groups: (1) Collaborative Care with Behavioural Activation plus Usual GP care intervention, or (2) Usual GP care. Participants will be randomised by the York Trials Unit Randomisation Service.

Participants randomised to the collaborative care intervention group will be initially contacted by a case manager to arrange their first session of collaborative care with BA. Future sessions may be carried out either face to face or over the phone for a period of 8-10 weeks. Participants in the control group will receive "usual care" under their GP. We will not interfere with usual GP care in the control arm and no treatment will be denied to patients through participating in this trial.

### Trial intervention

#### Intervention group: Collaborative care with behavioural activation

Participants randomised to the intervention group will receive low intensity collaborative care which has been designed specifically for those aged 75 or over with sub-threshold depression, over 8-10 weekly sessions. The defining features of collaborative care include a case manager working with the participant, with access to the GP and a mental health specialist. Collaborative care will be delivered by a case manager. If a case manager deems depression to have deteriorated (moving from sub-threshold to threshold) the participant will be referred back to their GP for appropriate management; the participant will be provided with the option of continuing to receive collaborative care. The additional elements of collaborative care include: telephone support; symptom monitoring and active surveillance (facilitated by computerised case management systems (PC-MIS); low intensity psycho-social management (BA). This will be delivered according to an established protocol [20,29]. Participants randomised to collaborative care will meet with a case manager for their first session, after this initial meeting subsequent sessions will be on a weekly basis either conducted face to face or by telephone according to patient preference.

#### Control group

Participants randomised to the control group will receive usual primary care management of sub-threshold depression offered by their GP, in line with NICE depression guidance as implemented by their GP and local service provision [5,6].

### Inclusion and exclusion criteria

Eligible participants will be identified from GP practice lists. The following eligibility criteria will be used:

Inclusion:

• Aged over 75

• Screen positive to at least 1 of the Whooley [21,24] questions and is classed as experiencing sub-threshold depression during diagnostic interview based on the M.I.N.I. (Mini-International Neuropsychiatric Interview) [26]

Exclusion:

• Screen positive but suffering with above threshold depression (Major Depressive Disorder) based on the M.I.N.I. [26]

• Known alcohol dependency (as recorded on GP records)

• Any known co-morbidity that would in the GP's opinion make entry to the trial inadvisable (e.g. recent evidence of self-harm, known current thoughts of self harm, significant cognitive impairment)

• Other factors that would make an invitation to participate in the trial inappropriate (e.g. recent bereavement; terminal malignancy)

• Known to be experiencing psychotic symptoms (as recorded on GP records)

### Sample size calculation

To detect a minimum effect size of 0.3, with 80% power and a two-sided 5% significance level would require 352 patients (176 in each group). Although this is an individually-randomised trial, there may be potential clustering at the level of each collaborative care case manager and hence we need to inflate the sample size to account for this. Based upon an ICC = 0.02 and a caseload size of 20, the design effect would be 1.38 (1 + (20 - 1) × 0.02) and we would require 486 patients (243 in each group). Allowing for a potential loss to follow-up of 10% the final sample size needed is 540 patients (270 in each group).

### Recruitment

Recruitment will take place through GP practices in primary care. GP Practices will be recruited to the study after a member of the study team has provided the practice with written information and visited the practice to explain the study and what participation would entail.

Potential participants who meet the initial inclusion criteria of being a registered patient at the participating practice and aged over 75 years will be identified by GP practices. The practice will be asked, at this stage, to screen out all patients using the exclusion criteria. All eligible patients will be sent a letter of invitation by the practice, they will be given the opportunity to decline participation but still provide some demographic information and reason for declining, in order to provide comparison information with those who are participating. All patients who consent to take part in the CASPER study at this stage will be part of the CASPER cohort.

### Randomisation

Participants with sub-threshold depression who meet the inclusion criteria and have provided written consent will be eligible for randomisation into the trial. Randomisation will be carried out by the York Trials Unit Randomisation Service using simple randomisation with a computer based algorithm. Randomisation will be carried out once all relevant data are collected and entered into the study database. Participants will be randomised on a 1:1 basis to either the intervention group or control group. Participants who meet the inclusion criteria but have below- or above-threshold depression will not be randomised, these participants will form the cohort.

### CASPER epidemiological cohort

Participants who return a completed consent form and baseline questionnaire will be eligible for inclusion in the CASPER cohort. This design has been termed the "cohort multiple randomised controlled trial (cmRCT)", [25] with the following design features: (I) Recruitment of a large observational cohort of patients with the condition of interest; (II) Regular measurement of outcomes for the whole cohort; (III) Capacity for multiple randomised controlled trials over time. Therefore, the design has the following advantages: ongoing information as to the natural history of the condition and to treatment as usual; and a facility for multiple randomised controlled trials. In this case, we are interested in following the natural history of depressive symptoms amongst older people; comparing health outcomes for older people with and without depressive symptoms and potentially in the future, using this cohort to recruit for future trials in this age group.

### Follow-up

Data collection will initially occur at five time points. Data will be collected at invitation, baseline (pre-randomisation/pre-assessment), diagnostic interview for participants entering the trial (pre-randomisation), at 4 months post-randomisation/post-assessment and 12 months post-randomisation/post-assessment for trial and cohort participants; additionally primary care sources will be checked for depression prescribing. With the exception of the diagnostic interviews and depression prescribing, all participants will be sent questionnaires by post at follow-up in order to collect self-report depression, quality of life, psychological anxiety and medication data. The same questionnaire will be used for trial and cohort participants.

#### Outcome measures

The primary outcome is depression severity and symptomatology at four months as assessed by the PHQ-9 on a continuous scale. Secondary outcomes include: depression severity and symptomatology (at 12 months), binary description of the PHQ-9 (at 4 & 12 months), quality of life measures (at 4 & 12 months), psychological anxiety (at 4 & 12 months), medication (at 4 & 12 months), and mortality (at 4 & 12 months).

Self-reported questionnaires data will be used to capture the following:

• Demographic details at invitation

• Whooley questions [21,24] at invitation and baseline

• Questions about physical health problems at baseline

• SF-12 [30] at baseline and follow-up

• EQ-5D [31] at baseline and follow-up

• GAD-7 [32] at baseline and follow-up

• Questions about falls at baseline and follow-up

• PHQ-9 [33] at baseline, diagnostic interview and follow-up

• PHQ-15 [34] at baseline and follow-up

• CD-RISC2 [35] at baseline and follow-up

Depression severity will be assessed by the PHQ-9. Quality of life will be assessed using the SF-12 and EQ-5D questionnaires. Psychological anxiety will be assessed using the GAD-7. All the above measures are completed by the participant; additionally, all participants will be asked if they have been diagnosed with any physical health problems. Depression medication data will be captured by self-report and directly from GP records, data will start to be collected from baseline onwards. Mortality will be established by flagging all randomised participants to the NHS Information Centre at regular intervals.

There are likely to be some cases of loss to follow-up due to death, this is likely to be around 8.2% *per annum *(calculated from national mortality rates for this age group). Loss to follow-up due to migration is unlikely as this group tends to be geographically stable and initial follow-up is only for 12 months. Where a participant has been lost to follow-up their data will be included in the main analysis up to where they have been lost to follow-up. Where a participant is lost to follow-up, efforts will be made to contact the participant.

### Data analysis

#### Analysis for validation of Whooley questions

The sensitivity, specificity and predictive values of the Whooley questions will be calculated with two-by-two contingency tables with the clinical diagnostic interview as the gold standard. Associated 95% confidence intervals will also be calculated for each estimate.

#### Statistical analysis

A linear regression model will be used to compare collaborative care with usual care on the primary outcome adjusted for baseline depression severity (as measured by the PHQ-9) and physical/functional limitations (as measured by the SF-12 physical functioning scale). To explore the potential clustering within collaborative care case managers the primary analysis will be repeated adjusting for the clustering using the Huber-White standard estimator (robust standard errors).

All secondary analyses will be conducted using linear or logistic regression, depending on the outcome measure, adjusting for similar covariates to the primary analysis. In addition, for each outcome measure the number of non-responders will be calculated for each treatment group and response rates compared. Appropriate sensitivity analyses will be used to examine the effects of missing data on outcomes.

### Withdrawal

Withdrawal can occur at any point during the study at the request of the participant. If a participant indicates they wish to withdraw from the study, withdrawal will be clarified as to whether the withdrawal is from the intervention, from follow-up or all aspects of the study. Where withdrawal is only from the intervention then follow-up data will continue to be collected. Data will be retained for all participants up to the date of withdrawal, unless they specifically request for their details to be removed.

### Economic analysis

Incremental cost-effectiveness analysis will be undertaken from a health and personal social services perspective following NICE guidance [36]. The economic analysis will estimate the value for money afforded by the collaborative care with behavioural activation and usual GP care intervention over and above usual GP care alone. QALYs will be estimated using the EQ-5D. This approach enables comparisons to be made across different health interventions and provides extra information for decision makers. QALYs will be estimated by measuring the area under the curve [37] which joins baseline and follow up EQ-5D utility scores derived from population based values.

Costs of the intervention, control and the total health care costs during the treatment and follow up period will be assessed. Individual take-up of depression management and control interventions will be measured and costs will be estimated using a bottom-up approach. Costs of the intervention, total health care costs and changes in outcome measures in the RCT will be combined to calculate the incremental cost-effectiveness ratios. The sensitivity of the cost-effectiveness ratio to different threshold values for a QALY will be demonstrated using cost-acceptability curves [38].

### Ethical Issues

NHS REC (National Health Service Research Ethics Committee) approval has been obtained from Leeds East REC (reference number 10/H1306/61) and local approvals have been obtained through local NHS R&D offices.

### Trial Management

The chief investigator (Simon Gilbody) will be in charge of the overall management of the trial. The York-based trial manager (Natasha Mitchell) will be responsible for the co-ordination of the study between sites. A trial co-ordinator and trial secretary will carry out the day to day activities involved in running the trial at each site. Delivery of collaborative care will be carried out by a dedicated & skilled case manager.

A local trial management group will be formed at each study centre and regular meetings will be held.

## Competing interests

The authors declare that they have no competing interests.

## Authors' contributions

All authors contributed to the design and development of the study protocol. NM & SG were responsible for writing this manuscript. All authors read and approved the final manuscript.
